# A case of virilization induced by a Krukenberg tumor from gastric cancer

**DOI:** 10.1186/1477-7819-6-19

**Published:** 2008-02-15

**Authors:** Matthias Hornung, Peter Vogel, Thomas Schubert, Hans-Jürgen Schlitt, Ulrich Bolder

**Affiliations:** 1 Department of Abdominal Surgery, University of Regensburg, 93053 Regensburg, Germany; 2 Department of Pathology, University of Regensburg, 93053 Regensburg, Germany

## Abstract

**Background:**

The Krukenberg tumor represents ovarian metastases associated with gastric cancer or other gastrointestinal malignancies. Histology shows typical mucus-production and numerous signet-ring cells. Occasionally Krukenberg tumors have endocrine function and, as a consequence, some patients demonstrate hirsutism and virilization.

**Case presentation:**

Here we report a case of virilization associated with an extensive gastric adenocarcinoma and Krukenberg tumor in a premenopausal woman. Virilization occurred three months after diagnosis of gastric cancer and the ovarian tumors. Palliative chemotherapy was initiated as primary therapy, but gastric outlet obstruction required a gastrojejunostomy. In addition, oopherectomy was performed to relieve abdominal tension and to abate hormonal effects. It is likely that virilization of the patient could have been prevented by earlier oopherectomy prior to development of hormone production.

**Conclusion:**

Despite the limitation in survival time early oopherectomy should be considered to prevent the development of virilization even in palliative situations if a Krukenberg tumor is diagnosed with gastric cancer.

## Background

Although incidence and mortality of gastric cancer have decreased over the last decades, it still remains the fourth most common cancer and the second leading cause of cancer-related death worldwide [1,2]. In some cases secondary tumor from gastric signet-cell adenocarcinoma appear in the ovaries. It was first described by Krukenberg in 1896 [3]. Histologically, Krukenberg tumors show diffuse stromal proliferation, mucus-production, and numerous signet-cells that usually can be found in both ovaries. In general, a mucus-producing gastric carcinoma with signet-cells in the stomach is diagnosed as a primary tumor. McGill *et al*. showed, that among 233 female patients with gastric cancer, there is an incidence of Krukenberg tumors of 18.2% in premenopausal women between 40 to 50 years-old, versus 0% in postmenopausal women [4]. Diagnosis of Krukenberg tumors represents advanced malignancy and there is still no effective therapy for this type of tumor. Therefore, prognosis is poor and median survival of patients ranges between 7 to 14 months [4,5].

Diagnosis is usually accomplished by CT scan or ultrasound [5]. The literature reveals several reports of pregnant women suffering from Krukenberg tumor in association with virilization or hirsutism. In these cases, both mother and infant suffer from the clinical signs of elevated androgen levels [6,7]. In this context one has to distinguish hirsutism, which describes an increase in body hair from virilization, with an additional change to male body features.

Here we report a case of a 41 years old female patient with Krukenberg tumor and strong signs of virilization without pregnancy. The case is of interest because it affected a non-pregnant patient and virilization occurred only three months after the initial diagnosis of Krukenberg tumor.

## Case presentation

**Figure 1:**
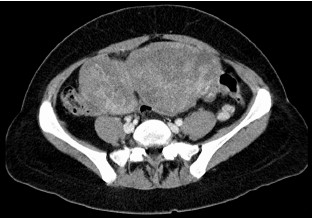
CT scan showing two very large ovarian tumors in the lower abdomen.

A 41-year-old woman presented to our surgical outpatient clinic with hypermenorrhea, followed by amenorrhea and discomfort in the upper abdomen with nausea and emesis. Gastroscopy and histology revealed a poorly differentiated primary gastric mucus-producing adenocarcinoma with numerous signet-ring cells in the distal corpus and the antrum. CT scan showed extensive tumor in the lower abdomen, which appeared to be an ovarian tumor or, as a differential diagnosis, a Krukenberg tumor (Figure 1). Endoscopic ultrasound of the tumor resulted in a uT3, N+ stage. Elevated tumor markers like CA 19-9 (817.7 U/ml, normal range: 0.0–37.0 U/ml), CA 72-4 (92.4 U/ml, normal range: <4.0 U/ml) and CA 125 (151.4 U/ml, normal range: <30.2 U/ml) were also present. Virilization was absent at the time of the initial diagnosis. Surgery was not initially considered due to the advanced stage of the primary tumor. Adjuvant chemotherapy was initiated and the patient received six cycles of epirubicin, cisplatin and 5-fluorouracil, each cycle one week after the ECF regimen [8].

**Figure 2:**
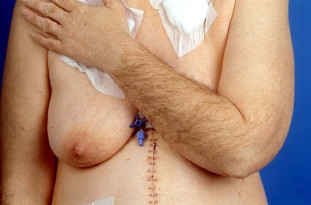
Extensive increase of body hair and muscle mass of the premenopausal patient.

**Figure 3:**
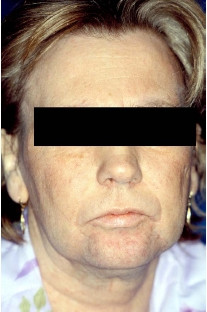
Androgenized facial features.

**Figure 4:**
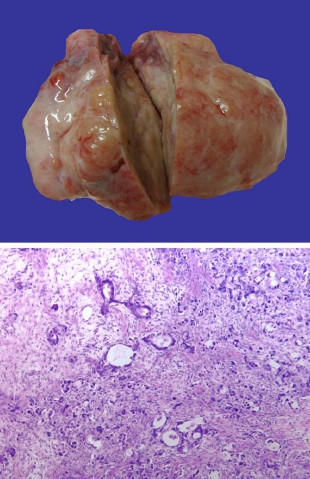
**A) Macroscopic view on the resected inhomogeneous tumor formations.** B) Histology revealed mucus-producing glandular structures, small solid nests and signet-ring cells surrounded by ovarian stroma.

Side effects of chemotherapy consisted of appetite reduction, nausea and emesis, and a weight loss of 10 kg within three months. In addition, the patient developed a marked increase in body hair covering of her arms, legs, and face. A notably deeper voice and an androgenized body feature, with increased muscle strength, were also observed (Figure 2 and 3). Gynecological examination revealed no clitoral enlargement. Circulating levels of testosterone (1.33 μg/l, normal range: <0.62 μg/l), progesterone (52.29 nmol/l, normal range: 0.64–2.58 nmol/l) and 17-OH-progesterone (14.96 μg/l, normal range: 0.40–1.02 μg/l) were elevated, while other hormones like estradiol, androstendion, follicle-stimulating hormone, luteinizing hormone, prolactin and dehydroepiandrosterone sulfate were in the normal range. Since the patient experienced increasing episodes of vomiting, a repeat gastroscopy was performed. An expanded stomach full of nutrients, due to gastric outlet obstruction, was found. An operative intervention to improve the patient's condition was scheduled. During explorative laparotomy, the two Krukenberg tumors displacing the small intestine were found. Tumors and ovaries were removed and intraoperative histology confirmed a Krukenberg tumor from a diffuse gastric carcinoma (Figure 4). In the upper abdomen an obstructive gastric carcinoma with a widespread peritoneal carcinomatosis was found. Since complete removal with R0 resection could not be achieved, a gastrojejunostomy bypassing the stenosis was constructed. The postoperative course was without complications and oral feeding was started successfully on the third day after surgery. Chemotherapy was reinitiated with a modified regimen using 5-fluorouracil (2000 mg/m^2^) and oxaliplatin (50 mg/m^2^), with a total of eight cycles in a weekly schedule. During the chemotherapy tumor markers dropped to 45.2 U/ml (CA 19-9), 13.7 U/ml (CA72-4) and 31.9 U/ml (CA 125). Expectedly, the hormone levels returned to normal levels. The patient survived for 6 more months before she died from tumor recurrence, without returning to her normal phenotype.

## Discussion

The Krukenberg tumor is an ovarian metastasis of a primary tumor derived from abdominal or retroperitoneal organs [9]. Two-thirds of primary tumors are found in the stomach. The appendix, colon, small intestine, rectum, gallbladder and urinary bladder have also been reported as a site of the original carcinoma [9]. Even intramucosal gastric cancer may lead to a Krukenberg tumor [10]. Kiyokawa *et al*. reported in an extensive review of 120 cases that two-thirds of the Krukenberg tumors were diagnosed at the same time as the primary carcinoma [11]. Our case parallels this experience, since diagnosis of the gastric tumor and the Krukenberg tumor were established by the same CT scan. On the other hand Schoenfeld *et al*. reported metachroneous occurrence of a Krukenberg tumor 8 years after subtotal gastrectomy for adenocarcinoma [12]. In our case simultanous diagnosis was facilitated by clinical symptoms, which hinted to both organs. These signs were nausea, vomiting and upper abdominal tension in combination with hypermenorrhea, followed by amenorrhea. However, only abdominal swelling and abdominal pain are reported as symptoms of Krukenberg tumors, whereas abnormal vaginal bleeding and amenorrhea occur in only 20% of patients [11]. Most patients with Krukenberg tumor are in the premenopausal period of life and at least two-thirds of the tumors are bilateral [4,11]. Only a small number of patients have endocrine manifestations, including virilization, hirsutism, breast soreness and swelling, postmenopausal vaginal bleeding, as well as endometrial hyperplasia [11].

It is still unclear why some ovarian metastases lead to androgenizing hormone production followed by hirsutism or even worse, virilization as presented in our case. From the clinical point of view it is important to recognize that patients undergoing virilization or hirsutism may suffer from a disturbed body constitution with the serious consequence of social isolation. Numerous reports of virilization and hirsutism of mother and infant in association with Krukenberg tumors during pregnancy have been published [6,7,13-20] (Table 1). However, there are no reports regarding the time course from the diagnosis of a Krukenberg tumor to the development of virilization or hirsutism. Furthermore, it is unknown what triggers the development from hirsutism to virilization, with associated changes of the female body image.

**Table 1:** Published cases of Krukenberg tumor with virilization. None of them reported time delay between diagnosis of Krukenberg tumor and virilization.

**Publication**	**Number of reported cases**	**Pregnancy**	**Year of Publication**
Interstitial hemorrhage and rupture of a Krukenberg tumor with virilism. *Wagner et al. *(21)	1	No	1950
Krukenberg tumor complicating pregnancy; report of a case with androgenic activity. *Fox et al. *(6)	1	Yes	1965
Virilization coexisting with Krukenberg tumor during pregnancy. *Spadoni et al. *(20)	1	Yes	1965
Gonadotropin-dependent Krukenberg tumor causing virilization during pregnancy. *Connor et al. *(19)	1	Yes	1968
Metabolism of testosterone by virilizing Krukenberg tumor of the ovary. *Ances at al. *(18)	1	No	1968
A case of Krukenberg tumor with virilization aspects. *Sani et al. *(22)	1	No	1977
Approach to the mechanism of androgen overproduction in a case of Krukenbery tumor responsible for virilization during pregnancy. *Forest et al. *(16)	1	Yes	1978
Long-interval masculinizing Krukenberg tumor of the ovary. *Schoenfeld et al. *(12)	1	No	1982
Clinical and ultrastructural findings of an androgenizing Krukenberg tumor in pregnancy. Silva et al. (15)	1	Yes	1988
Tubular Krukenberg tumor in pregnancy with virilization. *Fung et al. *(14)	1	Yes	1991
Krukenberg tumor in pregnancy with virilization. A case report. *De Palma et al. *(13)	1	Yes	1995
Krukenberg tumor during pregnancy with maternal and fetal virilization: a difficult diagnosis. A case report. *Vauthier-Brouzes et al. *(7)	1	Yes	1997
Krukenberg tumors of the ovary: a clinicopathologic analysis of 120 cases with emphasis on their variable pathologic manifestations. *Kiyokawa et al. *(11)	4	1 of 4	2006

Histologically the tumor consisted of mucus-producing glandular structures, small solid nests and numerous signet-ring cells surrounded by partly luteinized ovarian stroma. In immunohistochemical studies the tumor cells showed a strong expression of cytokeratin 7 and focal expression of cytokeratin 20 consistent with the diagnosis of gastric adenocarcinoma. The neuroendocrine markers CD56, chromogranin and synaptophysin were negative ruling out a neuroendocrine subpopulation of the tumor. In addition, Krukenberg tumors of patients with virilization reveal a stromal luteinization, whereas these microscopic findings can be confirmed in only 1 of 4 patients with hirsutism. Furthermore only 6 of 97 patients lacking stromal luteinization have been shown to have endocrine changes. Therefore, it appears that androgenizing hormone production requires development of luteinized stroma in Krukenberg tumors and it is likely that early ovarectomy can prevent virilization since spontaneous regression of virilization is a rare event [11].

## Conclusion

In the present case, virilization appeared only three months after the diagnosis of Krukenberg tumor. The case suggests that hormone production leading to virilization requires a minimum period of three months. We therefore propose that a timely control of androgenizing hormones should be performed in cases of Krukenberg tumor derived from gastric cancer. Due to the possibility of rapidly developing virilization, surgical resection of symptomatic and hormone producing tumors should be offered to patients even in a palliative setting.

## Competing interests

The author(s) declare that they have no competing interests.

## Authors' contributions

MH participated in writing the manuscript and interpretation of data, patient care, PV carried out the surgical procedure with UB, interpretation of data; TS carried out histological analyses, HJS interpretation of data, UB conceptual design, participated in writing of the manuscript and carried out the surgical procedure with PV. All authors read and approved the final manuscript.

## References

[B1] Edwards BK, Brown ML, Wingo PA, Howe HL, Ward E, Ries LA, Schrag D, Jamison PM, Jemal A, Wu XC, Friedman C, Harlan L, Warren J, Anderson RN, Pickle LW (2005). Annual report to the nation on the status of cancer, 1975-2002, featuring population-based trends in cancer treatment. J Natl Cancer Inst.

[B2] Parkin DM, Pisani P, Ferlay J (1999). Global cancer statistics. CA Cancer J Clin.

[B3] (1973). Classic pages in obstetrics and gynecology: Friedrich Ernst Krukenberg: Fibrosarcoma ovarii mucocellulare (carcinomatodes). Archiv fur Gynakologie, vol 50, pp. 287-321, 1896. Am J Obstet Gynecol.

[B4] McGill FM, Ritter DB, Rickard CS, Kaleya RN, Wadler S, Greston WM, O'Hanlan KA (1999). Krukenberg tumors: can management be improved?. Gynecol Obstet Invest.

[B5] Kim HK, Heo DS, Bang YJ, Kim NK (2001). Prognostic factors of Krukenberg's tumor. Gynecol Oncol.

[B6] FOX LP, STAMM WJ (1965). KRUKENBERG TUMOR COMPLICATING PREGNANCY; REPORT OF A CASE WITH ANDROGENIC ACTIVITY. Am J Obstet Gynecol.

[B7] Vauthier-Brouzes D, Vanna Lim-You K, Sebagh E, Lefebvre G, Darbois Y (1997). [Krukenberg tumor during pregnancy with maternal and fetal virilization: a difficult diagnosis. A case report]. J Gynecol Obstet Biol Reprod (Paris).

[B8] Findlay M, Cunningham D, Norman A, Mansi J, Nicolson M, Hickish T, Nicolson V, Nash A, Sacks N, Ford H, . (1994). A phase II study in advanced gastro-esophageal cancer using epirubicin and cisplatin in combination with continuous infusion 5-fluorouracil (ECF). Ann Oncol.

[B9] Hale RW (1968). Krukenberg tumor of the ovaries. A review of 81 records. Obstet Gynecol.

[B10] Kakushima N, Kamoshida T, Hirai S, Hotta S, Hirayama T, Yamada J, Ueda K, Sato M, Okumura M, Shimokama T, Oka Y (2003). Early gastric cancer with Krukenberg tumor and review of cases of intramucosal gastric cancers with Krukenberg tumor. J Gastroenterol.

[B11] Kiyokawa T, Young RH, Scully RE (2006). Krukenberg tumors of the ovary: a clinicopathologic analysis of 120 cases with emphasis on their variable pathologic manifestations. Am J Surg Pathol.

[B12] Schoenfeld A, Pistiner M, Pitlik S, Rosenfeld JB, Ovadia J (1982). Long-interval masculinizing Krukenberg tumor of the ovary. Eur J Obstet Gynecol Reprod Biol.

[B13] de Palma P, Wronski M, Bifernino V, Bovani I (1995). Krukenberg tumor in pregnancy with virilization. A case report. Eur J Gynaecol Oncol.

[B14] Fung MF, Vadas G, Lotocki R, Heywood M, Krepart G (1991). Tubular Krukenberg tumor in pregnancy with virilization. Gynecol Oncol.

[B15] Silva PD, Porto M, Moyer DL, Lobo RA (1988). Clinical and ultrastructural findings of an androgenizing Krukenberg tumor in pregnancy. Obstet Gynecol.

[B16] Forest MG, Orgiazzi J, Tranchant D, Mornex R, Bertrand J (1978). Approach to the mechanism of androgen overproduction in a case of Krukenbery tumor responsible for virilization during pregnancy. J Clin Endocrinol Metab.

[B17] Bell RJ (1977). Fetal virilisation due to maternal Krukenberg tumor. Lancet.

[B18] Ances IG, Ganis FM (1968). Metabolism of testosterone by virilizing Krukenberg tumor of the ovary. Am J Obstet Gynecol.

[B19] Connor TB, Ganis FM, Levin HS, Migeon CJ, Martin LG (1968). Gonadotropin-dependent Krukenberg tumor causing virilization during pregnancy. J Clin Endocrinol Metab.

[B20] SPADONI LR, LINDBERG MC, MOTTET NK, HERRMANN WL (1965). VIRILIZATION COEXISTING WITH KRUKENBERG TUMOR DURING PREGNANCY. Am J Obstet Gynecol.

[B21] WAGNER H (1950). [Interstitial hemorrhage and rupture of a Krukenberg tumor with virilism.]. Zentralbl Gynakol.

[B22] Sani G, Borghetti G, Bassani P, Turi A (1977). [A case of Krukenberg tumor with virilization aspects]. Minerva Ginecol.

